# Cytokines and Diabetes Research

**DOI:** 10.1155/2014/920613

**Published:** 2014-01-16

**Authors:** Jian Xiao, Ji Li, Lu Cai, Subrata Chakrabarti, Xiaokun Li

**Affiliations:** ^1^School of Pharmacy, Wenzhou Medical College, Wenzhou 325035, China; ^2^School of Medicine and Biomedical Sciences, University at Buffalo-SUNY, Buffalo, NY 14214, USA; ^3^School of Medicine, University of Louisville, Louisville, KY 40202, USA; ^4^London Health Science Center Pathology, University of Western Ontario, London, ON, Canada N6A 5A5

In recent years, the role of the inflammatory system in the pathogenesis of diabetes has been increasingly investigated. Cytokines, a group of proteins that are expressed by several cell types, act as immune mediators and regulators. Depending on the period of pregnancy, a predominant inflammatory profile is defined by increased production of cytokines. Insulin resistance has been associated with abnormal secretion of proinflammatory cytokines such as tumor necrosis factor-*α* (TNF-*α*) and Interleukin-6 (IL-6) and decreased production of anti-inflammatory mediators such as IL-4 and IL-10. Despite some controversies regarding specific cytokine levels, type 2 diabetes mellitus (T2DM) is currently regarded as a chronic inflammatory disease, while type 1 diabetes (T1D) is considered to be a T-helper-(Th)-1 autoimmune disease.

Extensive research in animals and in humans over the last decade has revealed important functions of cytokines in diabetes; adiponectin (APN) and leptin can decrease hepatic gluconeogenesis, resistin (REN) can increase hepatic gluconeogenesis and glycogenolysis, IL-6 can decrease glycogen synthesis, and TNF-*α* can decrease glucose uptake in liver. Both of them can block hepatic insulin signalling by interfection of insulin receptor signalling and insulin signal transduction. Thus, cytokines are involved in nearly every facet of immunity, inflammation, and development of diabetes.

In this special issue, we have invited some papers hoping to shed light on some aspects of this very interesting field. We have collected 7 papers by scientists from 5 countries. In the submitted research papers, Y. Li et al. summarize recent findings regarding the relationship between adipocytokines and hepatic insulin resistance. Excessive adipose tissue may be detrimental partially through secretion of the following cytokines: TNF-*α*, IL-6, and resistin. In contrast, the presence of adipose tissues is vital in the prevention of hepatic insulin resistance via secretion of the following cytokines: leptin and adiponectin. While J. Su and colleagues review the relationship between the endoplasmic reticulum (ER) and autophagy, inflammation, and apoptosis in DM to better understand the molecular mechanisms of diabetes, the authors suggest that the ER is therefore an attractive potential therapeutic target, and maintaining or improving ER function appropriately may prevent diabetes. Z. Meng et al. concluded that ethanol causes glucose intolerance by increasing hepatic expression of 11*β*-hydroxysteroid dehydrogenase type 1 (11*β*-HSD1) and glucocorticoid receptor (GR), which leads to increased expression of gluconeogenic and glycogenolytic enzymes. In the following papers, J. Liu et al. have shown that uncoupling proteins (UCPs) may affect the development of DM through decreasing mitochondrial membrane potential, increasing energy expenditure especially through glucose and lipid metabolisms, downregulating ROS generation, and gene polymorphisms. In a very interesting research paper, J. Vcelakova et al. have shown, that in T1D patients, important immune response-related pathways were involved. These important immune response-related processes largely included the induction of Th17 and Th22 responses, as well as cytoskeletal rearrangements, MHCII presentation, and the upregulation of CD4, TGF-beta, and STAT3. These findings potentially suggest that these processes could be utilised as predictive markers for the development of T1D or as molecular targets for the repression of specific immunocompetent cell populations for the treatment of diabetes. On the other hand, H. Meng and colleagues demonstrate that amyloid precursor protein 17 peptide (APP17 peptide) has a comprehensive therapeutic effect on diabetic encephalopathy, particularly through improving glycol metabolism. Finally, M. Cui et al. have shown that AMPK activation, which was represented by the level of p-AMPK, did not correlate with the improvement of metabolic conditions in diabetes mice, implying that AMPK activation may not participate in mediating the beneficial effects of chronic caloric restriction (CR) or exercise. However, the autophagy activity might be related to the improved metabolic conditions; thus autophagy may play a role in mediating the effects of chronic CR.

